# Autophagy-independent enhancing effects of Beclin 1 on cytotoxicity of ovarian cancer cells mediated by proteasome inhibitors

**DOI:** 10.1186/1471-2407-12-622

**Published:** 2012-12-27

**Authors:** Chuan Liu, Xu Yan, Hua-Qin Wang, Yan-Yan Gao, Juanjuan Liu, Zhenhua Hu, Dawo Liu, Jian Gao, Bei Lin

**Affiliations:** 1 Department of Obstetrics & Gynecology, Shengjing Hospital Affiliated to China Medical University, Shenyang, 110004, China; 2 Department of Prosthodontics, School of Stomatology, China Medical University, Shenyang, 110001, China; 3 Department of Biochemistry & Molecular Biology, China Medical University, Shenyang, 110001, China

**Keywords:** Proteasome inhibition, Beclin1, Ovarian cancer

## Abstract

**Background:**

The ubiquitin-proteasome system and macroautophagy (hereafter referred to autophagy) are two complementary pathways for protein degradation. Emerging evidence suggests that proteasome inhibition might be a promising approach for tumor therapy. Accumulating data suggest that autophagy is activated as a compensatory mechanism upon proteasome activity is impaired.

**Method:**

Autophagy activation was measured using acridine orange staining and LC3 transition. Cell viability and apoptosis were measured using MTT assay and flow cytometry, respectively. Beclin 1 expression vectors or shRNA against Beclin 1 (shBeclin 1) were transfected to investigate the role of Beclin 1 in autophagy activation and cytotoxicity of ovarian cancer cells induced by proteasome inhibitors.

**Results:**

Proteasome inhibitors suppressed proliferation and induced autophagy in ovarian cancer cells. Neither phosphoinositide 3-kinase (PI3K) inhibitors nor shRNA against Beclin 1 could abolish the formation of acidic vacuoles and the processing of LC3 induced by proteasome inhibitors. Moreover, Beclin 1 overexpression enhanced anti-proliferative effects of proteasome inhibitors in ovarian cancer cells.

**Conclusions:**

For the first time, the current study demonstrated that proteasome inhibitors induced PI3K and Beclin 1-independent autophagy in ovarian cancer cells. In addition, this study revealed autophagy-independent tumor suppressive effects of Beclin 1 in ovarian cancer cells.

## Background

The ubiquitin-proteasome system serves as a major intracellular pathway for protein degradation in mammalian cells [1]. Many proteins involved in cancer cell growth and survival are regulated by proteasomal degradation [2]. In this connection, proteasome inhibitors constitute a novel class of anti-tumor agents with pre-clinical and clinical evidence of activity against hematologic malignancies and solid tumors [3]. Macroautophagy (hereafter is referred as autophagy) is an evolutionarily conserved catabolic process by which cell destructs its cytoplasmic content and organelles through the lysosomal machinery [4]. Autophagy is initiated by the formation of a double-membrane bound vacuole (autophagosome), which sequesters cytosolic proteins and organelles such as mitochondria, endoplasmic reticulum. Autophagosomes are short-lived organelles that fuse with acidic lysosomes to produce autolysosomes where the sequestered content is degraded by lysosomal enzymes, and amino acids and sugars are recycled into the cytosol for reuse. Morphologically, autophagy is characterized by the formation of LC3+ double-membrane bound autophagosomes, the accumulation of acidic vesicular organelles and autolysosomes in the cytoplasm [5-7]. Autophagy was originally recognized as a crucial prosurvival mechanism to supply the cell with nutrients under unfavorable grown conditions [4]. It is now clear that autophagy plays a crucial role in development, programmed cell death and aging [4,8-10]. Dysregulation of autophagy has been involved in many human diseases including cancers. The fact that autophagy can have both suppressive and promoting roles in carcinogenesis makes it an attractive target in cancer research [10]. As a tumor suppressing mechanism, autophagy serves as an alternative to apoptosis to eliminate transformed cells [4]. Moreover, tumorigenesis is often associated with a reduced autophagy while genes that are involved in the execution of autophagy are found to be tumor suppressors [4]. On the other hand, autophagy may facilitate tumor growth and survival by providing tumor cells a selective advantage to therapy resistance and aggressiveness [4,10]. As two important intracellular pathways for protein degradation in mammalian cells, autophagy functions complementarily with the ubiquitin-proteasome system [1,11], and suppression of UPS can activate autophagy [12-20].

Emerging evidence shows that autophagy is important in the regulation of cancer development and progression [10]. However, the role of autophagy is complicated and autophagy may have opposing consequences in cells. On one hand, autophagy may protect tumor cells from nutrient deprivation and hypoxia; on the other hand, autophagy defect is associated with the development of cancer [8,21].

Beclin 1 is a tumor suppressor gene product that allosterically activates the class III phosphatidylinositol 3-kinase (PI3KC3), which is essential for the recruitment of other autophagy-related gene (Atg) proteins to the phagophore assembly site (PAS) to initiate autophagosome formation [22,23]. The BH3 binding groove of Bcl-XL/Bcl-2 binds the BH3 helix of Beclin1, preventing Beclin1 from recruitment to the PI3KC3 complex [24,25]. Recently, accumulating studies suggest that autophagy can also occur in a Beclin1-independent manner and in this case PI3K inhibitors fails to suppress it [26-30].

Here we reported that proteasome inhibitors induced cell death and autophagy in ovarian cancer cells. It was of note that MG132-induced autophagy was accompanied by a reduction of Beclin 1. In addition, we reported that proteasome inhibitors elicited autophagy even in shRNA against Beclin 1 (shBeclin 1) transfected cells, or in the presence of PI3Ks inhibitors, indicating that proteasome inhibitors caused Beclin 1/PI3Ks-independent autophagy. Furthermore, we demonstrated that Beclin 1 overexpression enhanced proteasome inhibitors-mediated cell death of ovarian cancer cells. Collectively, these data suggested that Beclin 1 sensitized ovarian cancer cells to proteasome inhibitors in an autophagy-independent manner.

## Methods

### Culture of multiple cancer cell lines

SKOV3, OVCAR3 and A2870 ovarian cancer cell lines were maintained in DMEM (Sigma-Aldrich, Saint Louis, MO) supplemented with 10% fetal bovine serum (FBS, Sigma-Aldrich, Saint Louis, MO).

### Chemicals

MG132, epoxomicin, PSI and lactacystin were purchased from Calbiochem (La Jolla, CA). 0.02% DMSO was used as vehicle control.

### Cell viability assays

For cell viability assays, cells were plated in 96-well dishes (1 × 10^4^ cells per well) and the next day were treated with or without apoptosis inducing agents in 10% FBS-containing media and grown over a 24-h period. Cell viability was assessed using the 3-(4,5-dimethylthiazol-2-thiazolyl)-2,5-diphenyl tetrazolium bromide (MTT) assay (Chemicon, Bedford, MA) according to the manufacturer’s instruction.

### Detection of apoptotic cell death

For cell death assays, cells were washed twice in phosphate-buffered saline and then stained with Annexin V-FITC (Biovision, Mountainview, CA) and propidium iodide (PI, Sigma-Aldrich) according to the manufacturer’s instructions. After staining with Annexin V-FITC and PI, samples were analyzed by fluorescence-activated cell scanner (FACScan) flow cytometer (Becton Dickinson, Franklin Lakes, NJ).

### Acridine orange staining for acidic vesicular organelles

Acridine orange was added at a final concentration of 1μg/ml for a period of 15 min. Pictures were obtained with a fluorescence microscope (Olympus) equipped with a digital camera (Olympus).

### Western blot analysis

Cells were lysed in lysis buffer (20 mM Tris–HCl, 150 mM NaCl, 2 mM EDTA, 1% Triton-X100 and protease inhibitor cocktail (Sigma-Aldrich, Saint Louis, MO). Cell extract protein amounts were quantified using the BSA protein assay kit. Equivalent amounts of protein (25 μg) were separated using 12% SDS-PAGE and transferred to PVDF membrane (Millipore Corporation, Billerica, MA).

### Caspase-3 activity assay

For caspases-3 enzymatic assays, 50 μg whole-cell extract was added to reaction buffer containing 25 mm HEPES (pH 7.5), 4 mm CHAPS, 1mm dithiothreitol (DTT), 1 mm phenylmethylsulfonyl fluoride (PMSF), 2 μg/ml aprotinin, 1 μg/ml leupeptin, and 2 μg/ml pepstatin, to achieve a total reaction volume of 500 μl. Ac-DEVD-AMC (Ac-Asp-Glu-Val-Asp-7-amino-4-methylcoumarin; Alexis Biochemicals, San Diego, CA) was added to the mixture at a concentration of 100 μM and incubated for 1 h at 37°C. Cleavage of the substrate was measured by fluorescence spectrometer (HTS 7000; PerkinElmer, Boston, MA) using an excitation and emission wavelength of 360 and 465 nm, respectively. The activities were expressed as fluorescence increase per microgram of protein.

### DNA construction and transfection

Beclin 1 plasmid was constructed by PCR and cloned into pcDNA3.1 vector. The construct was verified by DNA sequencing. Short hairpin RNA (shRNA) against Beclin 1 (shBeclin 1) or Atg7 (shAtg7) was purchased from Open Biosystems. Cells were transfected with Lipofectamine 2000 reagent (Invitrogene) as instructed by the supplier.

### Statistics

The statistical significance of the difference was analyzed by ANOVA and post hoc Dunnett’s test. Statistical significance was defined as p<0.05. All experiments were repeated three times, and data were expressed as the mean±SD (standard deviation) from a representative experiment.

## Results

### Proteasome inhibitors inhibited proliferation and induced apoptosis in ovarian cancer cells

**Figure 1:**
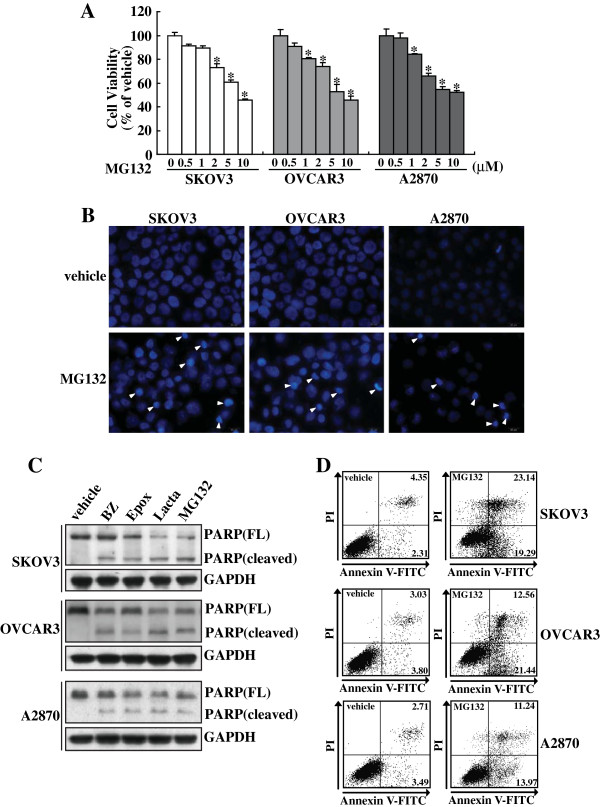
**Growth inhibition and apoptosis of ovarian cancer cells induced by proteasome inhibitors. A**, SKOV3, OVCAR3 or A2870 cells were treated with the indicated concentrations of MG132 for 24 h, and cell viability was measured using MTT assay. **B**, SKOV3, OVCAR3 or A2870 cells were treated with 5 μM of MG132 for 24 h, and nuclear morphology was analyzed using Hoechst 33258 staining. **C**, SKOV3, OVCAR3 or A2870 cells were treated with vehicle, bortezomib (BZ), epoxomicin (Epox), lactacystin (Lacta), or MG132 for 24 h, and Western blot analysis was performed using the indicated antibodies. **D**, SKOV3, OVCAR3 or A2870 cells were treated with the indicated concentrations of MG132 for 24 h, and apoptotic cells were measured using Annexin V and PI double staining followed by flow cytometry. *, *P*<0.01.

To study the effect of blockade of ubiquitin-proteasome system on proliferation of ovarian cancer cells, SKOV3, OVCAR3 and A2870 cells were treated with proteasome inhibitor MG132 at concentrations ranging from 0 to 10 μM for 24 h, the cell viability was determined using the MTT assay. MG132 significantly reduced cell proliferation in these cell lines in a concentration-dependent manner (Figure 1A). To determine the incidence of apoptosis morphologically, we stained the nuclei of 5 μM MG132 treated SKOV3, OVCAR3 and A2870 cells with Hoechst 33258. Apoptotic morphological characteristics such as chromatin condensation and nuclear fragmentation were detected in these ovarian cancer cells treated with 5 μM of MG132 (Figure 1B). Western blot confirmed that proteasome inhibitors including MG132, epoxomicin (Epox), Lactacystin (Lacta) and bortezomib (BZ) elicited cleavage of PARP in SKOV3, OVCAR3 and A2870 cells (Figure 1C). Annexin V-FITC and PI double staining followed by flowcytometry also confirmed that 5 μM of MG132 caused apoptosis of SKOV3, OVCAR3 and A2870 cells (Figure 1D). 

### Proteasome inhibitors induced autophagy in ovarian cancer cells

**Figure 2:**
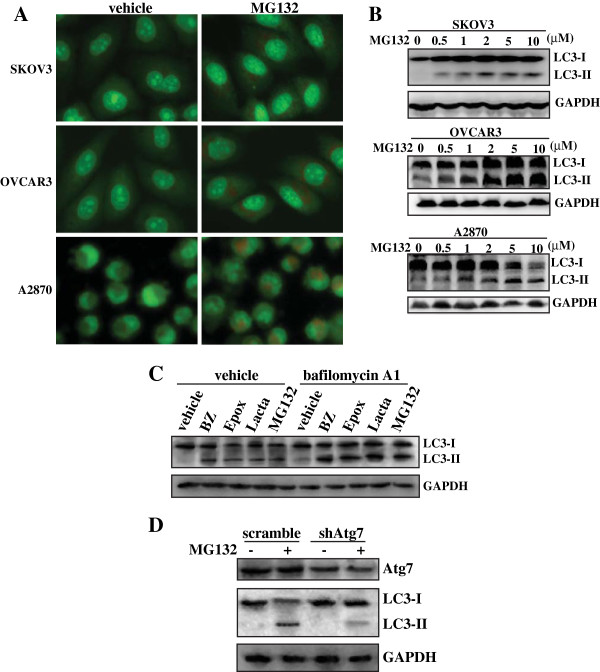
**Induction of autophagy by proteasome inhibitors in ovarian cancer cells. A**, SKOV3, OVCAR3 or A2870 cells were treated with vehicle or 5 μM of MG132 for 24 h, and the formation of acidic vacuoles were analyzed using AO staining. **B**, SKOV3, OVCAR3 or A2870 cells were treated with the indicated concentrations of MG132 for 24 h, Western blot analysis was performed to investigate LC3 transition. **C**, OVCAR3 were treated with vehicle, BZ, Epox, Lacta or MG132 in the absence or presence of bafiloymycin A1 for 24 h, and LC3 transition was measured using Western blot analysis. **D**, OVCAR3 were transfected with a scramble shRNA or shRNA specific against Atg7 (shAtg7) for 24 h, then treated with 5 μM of MG132 for additional 24 h, and Western blot was performed.

Under the light microscope, it was apparent that MG132 induced the formation of large vacuoles in the cytoplasm of ovarian cancer cells (data not shown). To determine the effect of MG132 on autophagy, we analyzed the accumulation of acidic vesicular organelles using the acridine orange (AO) staining. AO emitted bright red fluorescence in acidic vesicles but fluoresced green in cytoplasm and nucleus [6]. Vital staining of SKOV3, OVCAR3 or A2870 cells with AO revealed the appearance of acidic vesicular organelles after 5 μM of MG132 treatment (Figure 2A). Since the conversion of LC3 protein from LC3-I (the cytosolic form) to LC3-II (the membrane bound form) correlates with the extent of autophagy [5], we also analyzed the conversion of cytosolic LC3-I into LC3-II Western blot analysis. Results showed that MG132 induced LC3 transition in a dose-dependent manner in SKOV3, OVCAR3 or A2870 cells, respectively (Figure 2B). Similar like MG132, Western blot analysis also demonstrated that other proteasome inhibitors including BZ, Epox and Lacta also induced LC3 transition in OVCAR3 cells (Figure 2C). Since both autophagy induction and impaired autophagic degradation ascribes to accumulation of LC3-II [31], the effect of inhibiting lysosomal turnover of autophagosome contents by bafilomycin A1 [31] were also examined. Preventing lysosomal degradation by bafilomycin A1 cotreatment significantly increased LC3-II transition elicited by proteasome inhibitors (Figure 2C). In addition, knockdown of Atg7, a well-known autophagy essential gene, blocked LC3-II transition elicited by MG132 (Figure 2D). 

### Autophagy demonstrated little effects on MG132-mediated cytotoxicity in ovarian cancer cells

**Figure 3:**
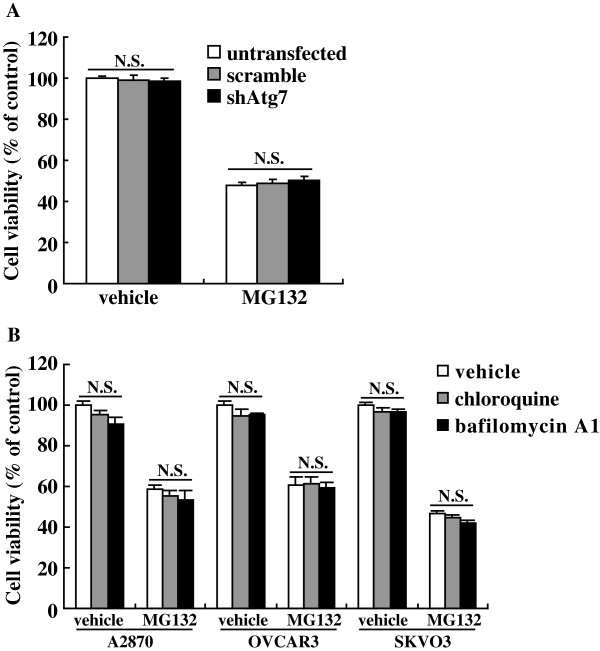
**Autophagy demonstrated little effects on cytotoxicity induced by MG132 in ovarian cancer cells. A**, OVCAR3 were transfected with scramble shRNA or shRNA specific against Atg7 (shAtg7) for 24 h, then treated with 5 μM of MG132 for additional 24 h, and cell viability was measured using MTT assay. **B**, SKOV3, OVCAR3 or A2870 cells were treated with 5 μM of MG132 in the presence of chloroquine or bafilomycin A1 for 24 h, and cell viability was measured using MTT assay. N.S., *p*>0.05.

To investigate the potential role of autophagy in cytotoxicity induced by proteasome inhibitors, OVCAR3 cells were transfected with shRNA against Atg7 (shAtg7) to suppress autophagy at the early stage. MTT assay demonstrated that shAtg7 demonstrated little effects on viability of OVCAR3 upon MG132 exposure (Figure 3A). Cotreatment with chloroquine or bafilomycin A1 to suppress autophagy at the late stage also demonstrated little effect on MG132-induced cytotoxicity of ovarian cancer cells (Figure 3B). 

### Wortmannin (WT) and 3-MA demonstrated no effect on proteasome inhibitors-induced autophagy in ovarian cancer cells

**Figure 4:**
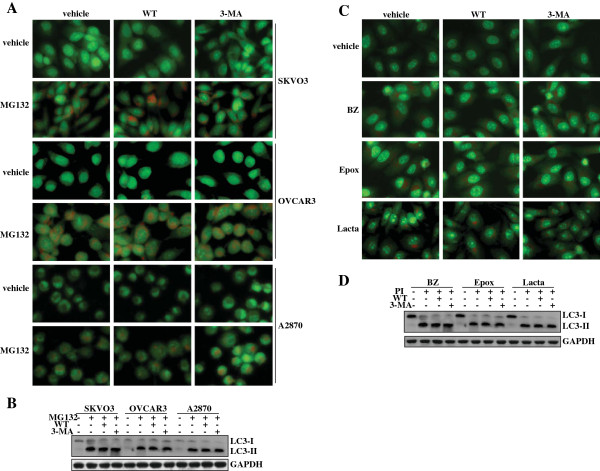
**PI3KC3-independent autopahgy induced by proteasome inhibitors in ovarian cancer cells. A**, Ovarian cancer cells were treated with 5 μM of MG132 in the absence or presence of wortmannin (WT) or 3-MA, and AVs formation was analyzed using AO staining. **B**, Ovarian cancer cells were treated with 5 μM of MG132 in the absence or presence of WT or 3-MA, and LC3 transition was analyzed using Western blot analysis. **C**, OVCAR3 cells were treated with the indicated proteasome inhibitor in the absence or presence of WT or 3-MA, and AVs formation was measured using AO staining. **D**, OVCAR3 cells were treated with the indicated proteasome inhibitor (PI) in the absence or presence of WT or 3-MA, and LC3 transition was analyzed using Western blot analysis.

To investigate the role of autophagy in proteasome inhibitors-mediated cytotoxicity of ovarian cancer cells, we managed to suppress autophagy activated by proteasome inhibitors using PI3Ks inhibitors. Unexpectedly, AO staining demonstrated that neither WT nor 3-MA suppressed MG132-induced accumulation of acid vacuoles in SKOV3, OVCAR3 and A2870 cells (Figure 4A). Western blot analysis confirmed that WT or 3-MA could not inhibit the conversion of LC3-I to LC3-II in MG132-treated SKOV3, OVCAR3 or A2870 cells (Figure 4B). To investigate whether proteasome inhibitors generally induced PI3K-independent autophagy, we tested some other proteasome inhibitors including BZ, Epox and Lacta in OVCAR3 cells. AO staining demonstrated that all these proteasome inhibitors induced accumulation of acidic vacuoles, neither WT nor 3-MA could block acidic vacuoles accumulation (Figure 4C). Western blot also confirmed that neither WT nor 3-MA could block transition of LC3-I to LC-II elicited by these proteasome inhibitors (Figure 4D). 

### Proteasome inhibitors elicited Beclin 1-independent autophagy in ovarian cancer cells

**Figure 5:**
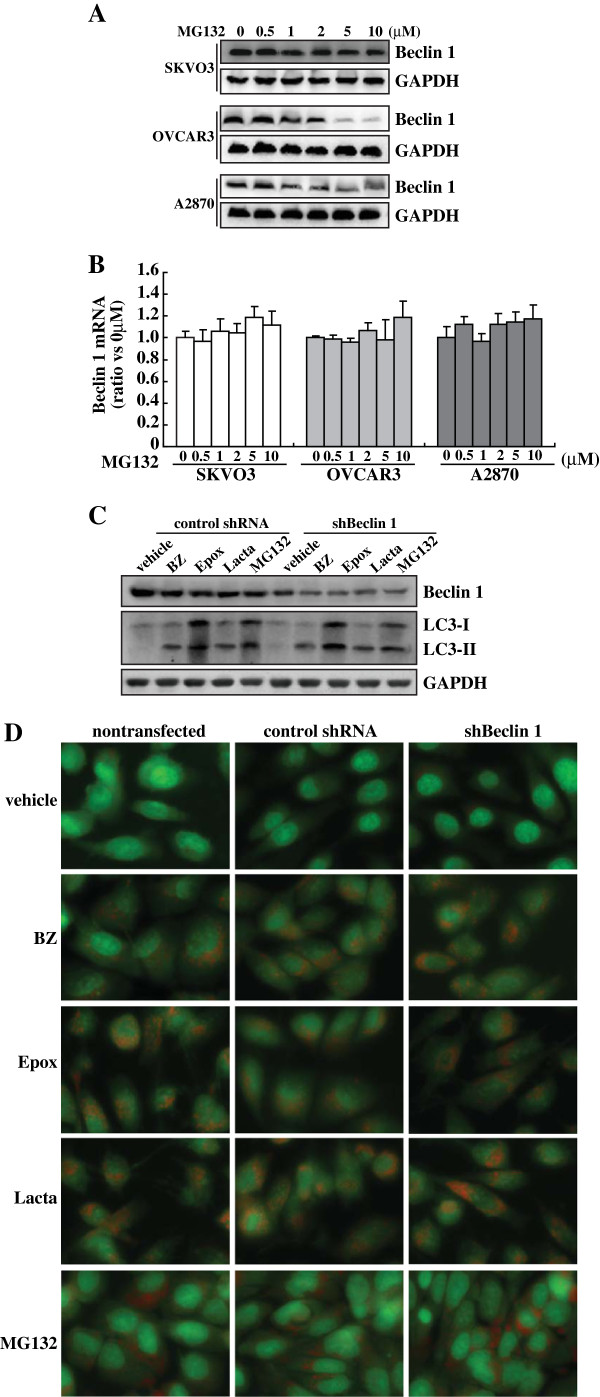
**Beclin 1-independent autophagy induced by proteasome inhibitors in ovarian cancer cells. A**, Ovarian cancer cells were treated with the indicated concentrations of MG132, and Western blot was performed using the indicated antibodies. **B**, Ovarian cancer cells were treated with the indicated concentrations of MG132, Beclin 1 mRNA was measured using real-time PCR. **C**, OVCAR3 cells were transfected with control shRNA or shRNA against Beclin 1 (shBeclin 1), then treated with the indicated proteasome inhibitor, and LC3 transition was analyzed using Western blot analysis. **D**, OVCAR3 cells were transfected with control shRNA or shRNA against Beclin 1 (shBeclin 1), then treated with the indicated proteasome inhibitor, and AVs formation was measured using AO staining.

As Beclin 1 is essential for the PI3K complex [32], observations that neither WT nor 3-MA was able to inhibit the increase in autophagosomes induced by proteasome inhibitors prompted us to confirm the role of Beclin 1 in proteasome inhibitors-induced autophagy. Western blot analysis demonstrated that MG132 reduced Beclin 1 expression in a dose-dependent manner in SKOV3, OVCAR3 and A2870 cells (Figure 5A). Real-time RT-PCR found that MG132 had no obvious effects on Beclin 1 mRNA expression (Figure 5B), suggesting that MG132 suppresses Beclin 1 at the translational or posttranslational level. To confirm the involvement of Beclin 1 in autophagy elicited by proteasome inhibition, Beclin 1 expression levels were further reduced by shRNA specific against Beclin 1 (shBeclin 1) in OVCAR3 cells. Western blot analysis confirmed that with some different extents, proteasome inhibitors reproducibly reduced Beclin 1 expression (Figure 5C). Specific shRNA against Beclin 1 (shBeclin 1) effectively reduced Beclin 1 levels under basal condition or upon exposure to proteasome inhibitors (Figure 5C). Importantly, transition of LC3-I to LC3-II (Figure 5C) and acidic vacuoles formation (Figure 5D) elicited by proteasome inhibitors was not affected by shBeclin 1.

### Overexpression of Beclin 1 enhanced cytotoxicity of ovarian cancer cells induced by proteasome inhibitors

**Figure 6:**
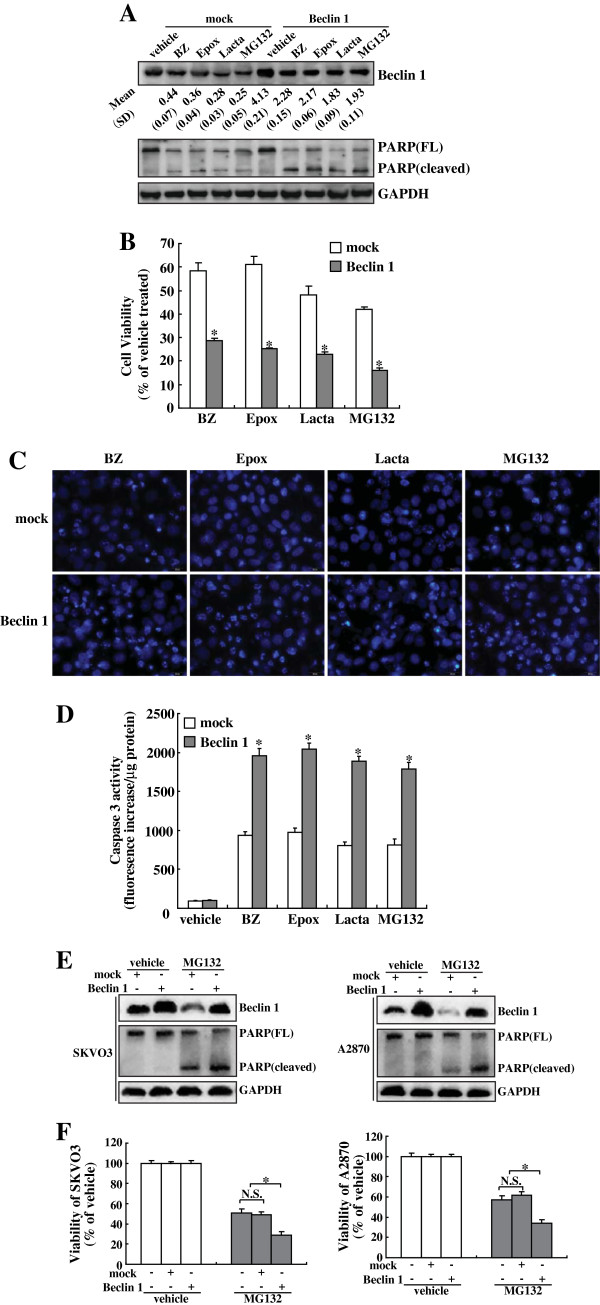
**Sensitizing effects of Beclin 1 overexpression on proteasome inhibitors-mediated cytotoxicity of ovarian cancer cells. A**, OVCAR3 cells were transfected with mock or Beclin 1 eukaryotic plasmid for 24 h, then treated with the indicated proteasome inhibitors for additional 24 h, and Western blot was performed using the indicated antibodies. A representative image was presented, and the ratios *vs* that of control (normalized by GAPDH) was noted at the bottom of the Beclin 1 image. **B**, OVCAR3 cells were transfected with mock or Beclin 1 eukaryotic plasmid for 24 h, then treated with the indicated proteasome inhibitors for additional 24 h, and cell viability was measured using MTT assay. **C**, OVCAR3 cells were transfected with mock or Beclin 1 eukaryotic plasmid for 24 h, then treated with the indicated proteasome inhibitors for additional 24 h, and nuclei morphology was measured using Hoechst 33258 staining. **D**, OVCAR3 cells were transfected with mock or Beclin 1 eukaryotic plasmid for 24 h, then treated with the indicated proteasome inhibitors for additional 24 h, and caspase-3 activity was measured. **E**, SKOV3 and A2870 cells were transfected with mock or Beclin 1 eukaryotic plasmid for 24 h, then treated with 5 μM of MG132 for additional 24 h, and Western blot was performed. **F**, SKOV3 and A2870 cells were transfected with mock or Beclin 1 eukaryotic plasmid for 24 h, then treated with 5 μM of MG132 for additional 24 h, and cell viability was analyzed using MTT assay. *, *P*<0.01.

To determine the influence of Beclin 1 in cytotoxicity of ovarian cancer cells induced by proteasome inhibitors, OVCAR3 cells were transfected with Beclin 1 eukaryotic expression vector. Compared to parental and pcDNA3.1 vector-transfected controls, a higher expression of Beclin 1 protein was detected in the Beclin 1-transfected OVCAR3 cells, and reduction of Beclin1 protein by proteasome inhibitors was suppressed by Beclin 1 transfection (Figure 6A). Overexpression of Beclin 1 significantly enhanced proteasome inhibitors-induced cytotoxicity of ovarian cancer cells, as assessed by cleavage of PARP (Figure 6A), MTT assay (Figure 6B), nuclei staining with Hoechst 33258 (Figure 6C), and caspase 3 activity assay (Figure 6D). In addition, analysis of PARP cleavage (Figure 6E) and MTT assay (Figure 6F) demonstrated that Beclin 1 overexpression also increased MG132-induced cytotoxicity of SKOV3 cells and A2870 cells. 

## Discussion

Two major proteolytic systems for the clearance of proteins are conserved in eukaryotic cells: the first is the UPS and the second is autophagy. Both UPS and autophagy are involved in most aspects of normal physiology and development. They are also implicated in multiple pathological states, such as cancer, neurodegeneration and aging. Although UPS and autophagy are generally thought to be independent from each other, recent investigations now support that the two proteolytic systems are functionally linked, and autophagy is activated and plays a compensatory role when UPS function is impaired [12-20]. Autophagy is frequently activated in cancer cells in response to chemo- or radiotherapy [33,34]. The contribution of autophagy to cell death induced by therapy generally remains controversial as autophagy protects some cancers against chemotherapy yet sensitizes others to chemotherapy-mediated cytotoxicity [33,34]. In the current study, we confirmed that proteasome inhibitors activated autophagy in ovarian cancer cells, as evidenced by accumulation of acidic vacuoles, increase in LC3-II transition. However, suppression of autophagy at the early stage by knockdown of Atg7, as well as at the late stage by cotreatment with chroroquine or bafilomycin A1 demonstrated little effects on cytotoxicity of ovarian cancer cells mediated by proteasome inhibition. The different role of autophagy in chemotherapy-induced cytotoxicity might represent cell-specific and/or stress-specific response. The dual roles of autophagy in survival and cell death require further clarification.

Beclin 1, the mammalian homologue of the yeast Atg6 was initially identified as a Bcl2-interacting tumor suppressor [35]. It is now known that Beclin 1 cooperates with several cofactors to activate lipid kinase PI3KC3, which is essential for recruitment of other Atg proteins to form autophagic vacuoles or autophagosomes [32]. However, several recent studies have demonstrated that some stimuli can also induce PI3KC3 and Beclin 1-independent autophagy, so named as non-canonical autophagy [26-30]. For example, resveratrol, Parkinsonian neurotoxin MPP^+^ and a small compound targeting the BH3 binding groove of Bcl-XL has been shown to activate autophagy in a Beclin 1-independent manner in breast cancer MCF7 cells, neuroblastoma cells and HeLa cells, respectively [26-30]. In the current study, for the first time, we reported that proteasome inhibitors elicited PI3KC3 and Beclin 1-independent autophagy in ovarian cancer cells, as evidenced by neither PI3Ks inhibitor wortmannin or 3-MA, nor shRNA against Beclin 1 could block accumulation of acidic vacuoles and increase in LC3-II transition induced by proteasome inhibitors. The mechanisms by which autophagosome formation can bypass the Beclin 1-PI3KC3 pathway remain to be clarified in the future.

Genetic analysis has revealed that Beclin 1 is implicated in tumorigenesis and plays a role in cellular proliferation [8,22,36,37]. It has been reported that overexpression of Beclin 1 activates autophagy and reduces the tumorigenetic potential of breast cancer cells [22]. In addition, overexpression of Beclin 1 has been shown to enhance the sensitivity of cervix and gastric cancer cells to chemotherapeutic drugs [38,39]. On the contrary, heterozygous disruption of Beclin 1 in mice increases cellular proliferation and results in spontaneous malignancies [37]. Consistent with previous reports [38,39], in the current study, flowcytometry analysis and caspase 3 activity assay indicated that a greater in crease in apoptosis was observed in Beclin 1-transfected cells than the mock-transfected cells. Controversially, Beclin 1 knockdown has been shown to promote apoptosis induced by doxorubicin in HepG2 cells [40]. These reports therefore suggest that Beclin 1 may modulate apoptosis in cell-specific and stimuli-specific patterns. It has been generally believed that Beclin 1 functions as a haploinsufficient tumor suppressor via autophagy activation [22,41]. However, in the current study, we found that proteasome inhibitors activated autophagy in a Beclin 1-independent manner. In addition, suppression of autophagy both at the early stage and at the late stage had no obvious effects on cytotoxicity mediated by proteasome inhibitors. On the contrary, Beclin 1 overexpression enhanced responsiveness of ovarian cancer cells to proteasome inhibitors-mediated cytotoxicity, indicating that Beclin 1 exerts autophagy-independent tumor suppressive effect in ovarian cancer cells upon exposure to proteasome inhibitors. Therefore, mechanisms underlying enhancing effects of Beclin 1 on chemosensitivity may be multifactorial, and the mechanisms by which Beclin 1 sensitizes ovarian caner cells to proteasome inhibition require further investigation.

## Conclusions

Proteasome inhibitors elicit PI3KC3 and Beclin 1 independent autophagy in ovarian cancer cells. In addition, Beclin 1 sensitizes ovarian cancer cells to proteasome inhibition in autophagy-independent manner.

## Competing interests

The authors declare that have no competing interests.

## Authors’ contributions

CL carried out the molecular genetic studies, cell culture, and participated in the data analysis. HQW conceived of the study, and participated in manuscript drafting. XY carried out transfection and cell culture. YYG carried out the DNA cloning and flow cytometry. JL and ZH participated in the cell culture. DL and JG participated in MTT assay and AO staining. BL conceived of the study, and participated in manuscript drafting and coordinate. All authors read and approved the final manuscript.

## Pre-publication history

The pre-publication history for this paper can be accessed here:

http://www.biomedcentral.com/1471-2407/12/622/prepub
